# Bcl-xL DNAzymes promote radiosensitivity and chemosensitivity in colorectal cancer cells via enhancing apoptosis

**DOI:** 10.1186/s40360-022-00553-x

**Published:** 2022-02-05

**Authors:** Zhen Yu, Jun Guo, Tao Meng, Lei Ge, Lin Liu, Haijiang Wang, Xinhui Yang

**Affiliations:** 1 Department of Gastrointestinal Surgery, The 3rd Affiliated Teaching Hospital of Xinjiang Medical University (Affiliated Cancer Hospital), Urumqi, 830010 P. R. China; 2 Department of Institute for Cancer Research, The 3rd Affiliated Teaching Hospital of Xinjiang Medical University (Affiliated Cancer Hospital), Urumqi, 830010 P. R. China

**Keywords:** DNAzyme, Bcl-xL, Radiotherapy, 5-fluorouracil, Colorectal cancer cells

## Abstract

**Background:**

RNA-cleaving deoxyribozymes (DNAzymes) are catalytic deoxyribonucleic acid molecules that have become a promising new class of gene suppressors by binding and cleaving target mRNA. This study investigated whether DNAzymes targeting Bcl-xL enhanced the effectiveness of radiotherapy and chemotherapy in colorectal cancer (CRC) cells.

**Methods:**

Two types of CRC cells, SW480 and SW837, were transfected with five DNAzymes. Cell viability, Bcl-xL expression and apoptosis were examined. SW480 xenograft model was used to examine the combined effects of Bcl-xL DNAzymes and 5-FU (or X-rays) on tumor growth.

**Results:**

Three Bcl-xL DNAzymes, DT882, DT883, and DT884 were identified to be effective in suppressing Bcl-xL expression and causing cell apoptosis. Furthermore, DT882 combined with 5-FU or radiotherapy addictively promoted cell apoptosis and significantly inhibited the growth of SW480 xenografts *in vivo*.

**Conclusions:**

These results suggest that Bcl-xL DNAzymes can enhance the radiosensitivity and chemosensitivity in CRC cells via inducing apoptosis.

**Supplementary Information:**

The online version contains supplementary material available at 10.1186/s40360-022-00553-x.

## Background

Colorectal cancer (CRC) is the third most common malignant tumor worldwide with 1.8 million new cases in 2018, and the fourth most common cause of cancer death [1]. At present, CRC is mainly treated by surgery, chemotherapy and radiation therapy. For rectal cancer, radiotherapy is a more common treatment and can be used before or/and after surgery along with chemotherapy. It is not common to use radiotherapy to treat colon cancer. However, radiotherapy may be used before surgery (along with chemotherapy) to shrink the tumor and to make it easier to remove [2]. 5-fluorouracil (5-FU), a kind of antimetabolite drugs targeting the metabolism of RNA bases, and radiotherapy (ionizing radiation) are widely used as first-line treatments for CRC [3–5]. However, cancer cells tend to be resistant to both 5-FU and radiotherapy due to the disorders of apoptosis signalling pathways [6, 7].

B-cell lymphoma-extra large (Bcl-xL) is a transmembrane molecule in the mitochondria, and belongs to the Bcl-2 family [8]. Bcl-xL prevents apoptosis by regulating the mitochondrial membrane permeability and inhibiting the release of cytochrome c [9]. It has been revealed that Bcl-xL is highly expressed in CRC tissues, and positively correlates with poorer overall patient survival [10, 11]. These results suggest that high expression of Bcl-xL may induce therapy resistance in CRC. Therefore, the development of drugs targeting Bcl-xL would contribute to increase the effectiveness of radiotherapy and chemotherapy.

Deoxyribozymes (DNAzymes) are single stranded DNA oligonucleotides capable of catalyzing chemical reactions [12, 13]. There have been no natural DNAzymes so far. However, DNAzymes can be isolated from *in vitro* selections or obtained by chemical synthesis [14, 15]. RNA-cleaving DNAzymes, catalyzing the cleavage of a ribonucleotide phosphodiester bond, are the most studied types, and can be used to specifically down-regulate the expression of target genes [16–18]. Our previous studies have demonstrated that DNAzymes targeting Bcl-xL induce apoptosis in prostate cancer PC3 cells, and enhance the therapeutic effects of chemotherapy [19, 20]. However, there have been no studies regarding the therapeutic effects of DNAzymes targeting Bcl-xL on CRC. In this study, we set out to examine the effects of DNAzymes targeting Bcl-xL on apoptosis in CRC cells, and to investigate whether the DNAzymes could improve the therapeutic effects of radiotherapy and chemotherapy.

## Methods

### Cell culture

CRC cell lines SW837 and SW480, established from the human adenocarcinomas of rectum and colon respectively, were used in this study. The two cell lines were purchased from the Cell Bank of Type Culture Collection (Shanghai, China), and maintained by L-15 medium (Gibco, Grand Island, NY, USA) supplemented with 10% fetal bovine serum (Gibco, Grand Island, NY, USA), 2 mM L-glutamine, and 1% penicillin/streptomycin (Beyotime, Shanghai, China). For 5-FU (CSNpharm, Shanghai, China) treatment, the cells were treated at 5 mg/mL, 10 mg/mL and 20 mg/mL respectively for 48 h. 20 mg/mL was selected for further studies. For radiotherapy group, the cells were irradiated at 2 Gy, 4 Gy, 6 and 8 Gy respectively. The X-rays were produced by an X-ray linear accelerator at a dose rate of 1.15 Gy/min (160 kV, 25 mA; RadSource, Suwanee, GA, USA). 48 h after irradiation, the cells were harvested for further studies.

### DNAzymes transfection

According to our previous study, we used 5 active DNAzymes, which were proved to selectively and effectively reduce Bcl-xL expression [19]. The 5 active DNAzymes were: DT867, 5’TTCCACGCAGGCTAGCTACAACGAAGTGCCCCG3’; DT880, 5’ ACAAAAGTAGGCTAGCTACAACGACCCAGCCGC3’; DT882, 5’ TTTTTATAAGGCTAGCTACAACGAAGGGATGGG3’; DT883, 5’ ACATTTTTAGGCTAGCTACAACGAAATAGGGAT3’; DT884, 5’TCTGAGACAGGCTAGCTACAACGATTTTATAAT3’. A scrambled DNA sequence was used as a negative control. For transfection, the cells were transfected with DNAzymes through Lipofectamine 2000 (Invitrogen, Carlsbad, CA, USA) according to the manufacturer’s instructions.

### Cell viability assay

Cell viability was measured by MTT method. The cells were seeded in a 96 well plate. 20 µL of MTT was added to each well and incubated for 4 h at 37^o^C. Then, the medium was replaced by 150 µL DMSO in each well to dissolve the MTT formazan. The absorbance was measured at 492 nm by a microplate reader (Thermo Fisher Scientific, Vantaa, Finland). Data were expressed as viability-inhibition rate.

### qPCR assay

Total RNA was extracted using TRIzol (Invitrogen, Gaithersburg, MD, USA) and was reversely transcribed using a RevertAid First Strand cDNA Synthesis Kit (Thermo Fisher Scientific, Carlsbad, CA, USA) in accordance with the kit instructions. Quantitative PCR was performed on an ABI 7500 Real Time PCR System (Applied Biosystems, Foster city, CA, USA) using a SYBR Green qPCR kit (Thermo Fisher Scientific, Vilnius, Lithuania). qPCR was performed using the following primers: β-actin, Forward 5‘CAACCGCGAGAAGATGACCCAGAT3’, Reverse 5’ ACGGCCAGAGGCGTACAGGGAT3’; Bcl-xl, Forward 5‘ACTTACCTGAATGACCACCTAGAGCC3’, Reverse 5’ GAAGAGTGAGCCCAGCAGAACC3’. The data were calibrated to β-actin and analyzed via the 2^−ΔΔCt^ method.

### Immunoblot assay

Total proteins from cells were extracted with RIPA lysis buffer (Beyotime, Shanghai, China), separated on 10% SDS-PAGE, and transferred to a polyvinylidene fluoride (PVDF) membrane. The membranes were cut into appropriate sizes according to the protein molecular weight, and incubated at 4^o^C overnight with the following primary antibodies: Bcl-xL (Abcam, Cambridge, UK) and β-actin (Abcam, Cambridge, UK). Then, the membrane was incubated with horseradish peroxidase (HRP)-labelled secondary antibody at room temperature for 2 h. After that, the band was detected with ECL reagents (7sea biotech, Shanghai, China). β-actin was used as an internal control.

### Apoptosis assay

Cell apoptosis was measured using an annexin V-fluorescein isothiocyanate/propidium iodide (Annexin-V FITC/PI) double-staining kit (BestBio, Shanghai, China). Briefly, the cells were digested using trypsin and were resuspended in binding buffer mixed with Annexin V-FITC solution. After 15 min, PI was added and incubated for 15 min. Cell apoptosis was detected and analyzed using a flow cytometer (BD Biosciences, San Diego, CA, USA).

###  *In vivo* xenograft tumor growth

6-week-old female BALB/c athymic nude mice (CAVENS Laboratory Animal Co. Ltd. Shanghai, China) were raised in the laboratory animal center of Xinjiang Medical University. The mice were fed with standard pellet diet and distilled water ad libitum, and were housed 5 mice/cage in a 12 h light/dark cycle, at a temperature of 23-25^o^C and a humidity of 40-60%. After acclimation, 1 × 10^6^ SW480 cells were subcutaneously injected into the abdominal wall. The tumor size was calculated by the formula: volume = length × width × width ×0.5. When the tumor size reached 70 mm^3^, the xenografts were treated with DNAzymes, 5-FU, radiotherapy or their combinations. For DNAzymes treatment, the mice were treated 2 times per week for 6 weeks by the way of center-intratumoral direct injection at a dose of 4 µM of DNAzymes mixed with Lipofectamine 2000. For 5-FU treatment, 5-FU (30 mg/kg) was injected intraperitoneally 2 times per week for 6 weeks [21, 22]. The prepared DNAzyme-Lipofectamine mixture (4 µM) was directly injected into the xenografts to enhance local antitumor activity. Control mice received the equivalent vehicle. For radiotherapy, xenografts were irradiated in fractions of 2 Gy/fraction given in 4 fractions per week. The X-rays were produced by an X-ray linear accelerator at a dose rate of 1.15 Gy/min (160 kV, 25 mA; RadSource, Suwanee, GA, USA). 2 Gy per fraction was used to simulate clinical radiotherapy setting. The mice were subjected to irradiation restricted to the xenografts, while the rest of the body was protected by a lead shield. After the last injection, all mice were sacrificed by cervical dislocation which applies pressure to the neck and dislocating the spinal column from the brain. The xenografted tumors were excised, fixed in 4% paraformaldehyde for 2 days, processed for paraffin sections, and stained with hematoxylin and eosin (H&E). Animal studies were approved by the Animal Ethics and Welfare Committee of Xinjiang Medical University. The experimental steps complied with the Chinese National Guidelines for the ethical review of laboratory animal welfare.

### Statistical analysis

Cellular experiments were performed in triplicate. Mouse experiments were performed using 6 mice per group. Data were expressed as mean ± standard deviation (SD) and were statistically analyzed by one-way ANOVA with Newman–Keuls, which was performed using GraphPad Prism software (La Jolla, CA, USA). *P* value less than 0.05 was considered statistically significant.

## Results

### Bcl-xL DNAzymes inhibited cell viability in SW480 and SW837 cells

**Fig. 1:**
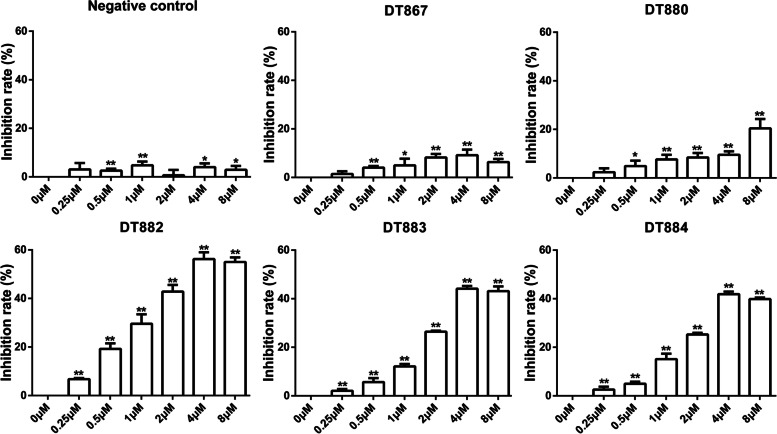
Cytotoxic effects of Bcl-xL DNAzymes on SW480 cells. SW480 cells were treated with different DNAzymes targeting Bcl-xL, DT867, DT880, DT882, DT883, and DT884, for 48 h. Cell viability was detected by MTT method. A scrambled DNA sequence was used as a negative control. n = 3. Data were presented as the mean ± SD. *P<0.05, **P<0.01 vs. 0 µM. NC, negative control

**Fig. 2:**
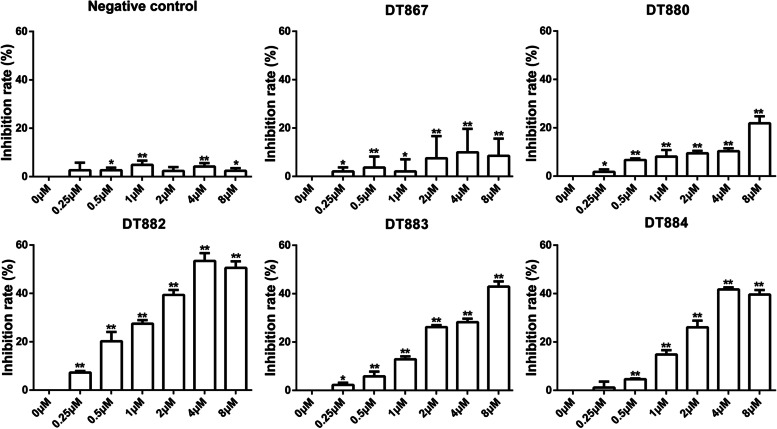
Cytotoxic effects of Bcl-xL DNAzymes on SW837 cells. SW837 cells were treated with different DNAzymes targeting Bcl-xL, DT867, DT880, DT882, DT883, and DT884, for 48 h. Cell viability was detected by MTT method. A scrambled DNA sequence was used as a negative control. n = 3. Data were presented as the mean ± SD. *P<0.05, **P<0.01 vs. 0 µM. NC, negative control

As a pro-survival factor, Bcl-xL is highly expressed in CRC cells. Downregulation of Bcl-xL by DNAzymes might reduce cell viability. We first examined whether Bcl-xL DNAzymes had cytotoxic effects on CRC cells. Human CRC cells SW480 and SW837 were transfected with different Bcl-xL DNAzymes, including DT867, DT880, DT882, DT883, and DT884. After 48 h, MTT assay results showed that all the Bcl-xL DNAzymes used in this study inhibited SW480 cell viability. The inhibitory effects of DT880, DT882, DT883, and DT884, were gradually increased with the increase of dose. The inhibition rate of DT886 at 8 µM was slightly lower than at 4 µM (Fig. 1). For SW837 cells, Bcl-xL DNAzymes, DT880, DT882, DT883, and DT884, increased the inhibition rate of cell viability in a dose-dependent manner (Fig. 2). The cells transfected with a scrambled DNA sequence (negative control) only exhibited less than 5% inhibitory rate of cell viability, which might be due to the cytotoxic effects of the transfection agent (Figs. 1 and 2). Taken together, these results suggest that Bcl-xL DNAzymes can inhibit cell viability in CRC cells.

### Bcl‑xL DNAzymes inhibited Bcl‑xL expression and caused apoptosis in SW480 and SW837 cells

**Fig. 3:**
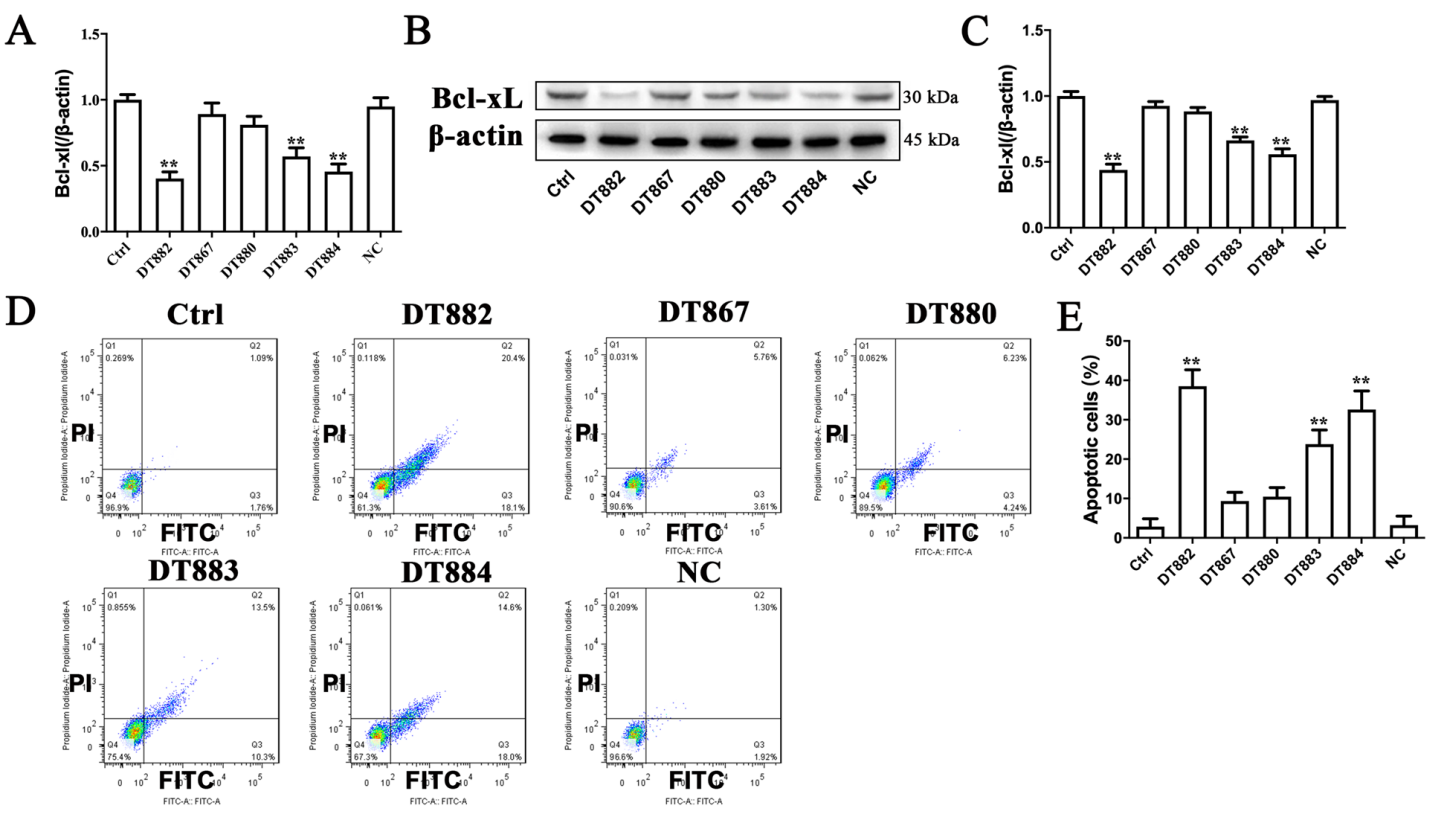
Effects of Bcl‑xL DNAzymes on Bcl‑xL expression and apoptosis in SW480 cells. After SW480 cells were treated with 4 µM Bcl‑xL DNAzymes for 48 h, Bcl‑xL expression was examined at mRNA (**A**) and protein (**B**-**C**) levels respectively. Apoptosis was detected by Annexin V-FITC/PI assay (**D**), and was expressed as percentages of apoptotic cells (**E**). n = 3. Data were presented as the mean ± SD. *P<0.05, **P<0.01 vs. Ctrl. The cells transfected with a scrambled DNA sequence were negative group (NC). The control cells (Ctrl) did not receive any treatment. Ctrl, control; NC, negative control

**Fig. 4:**
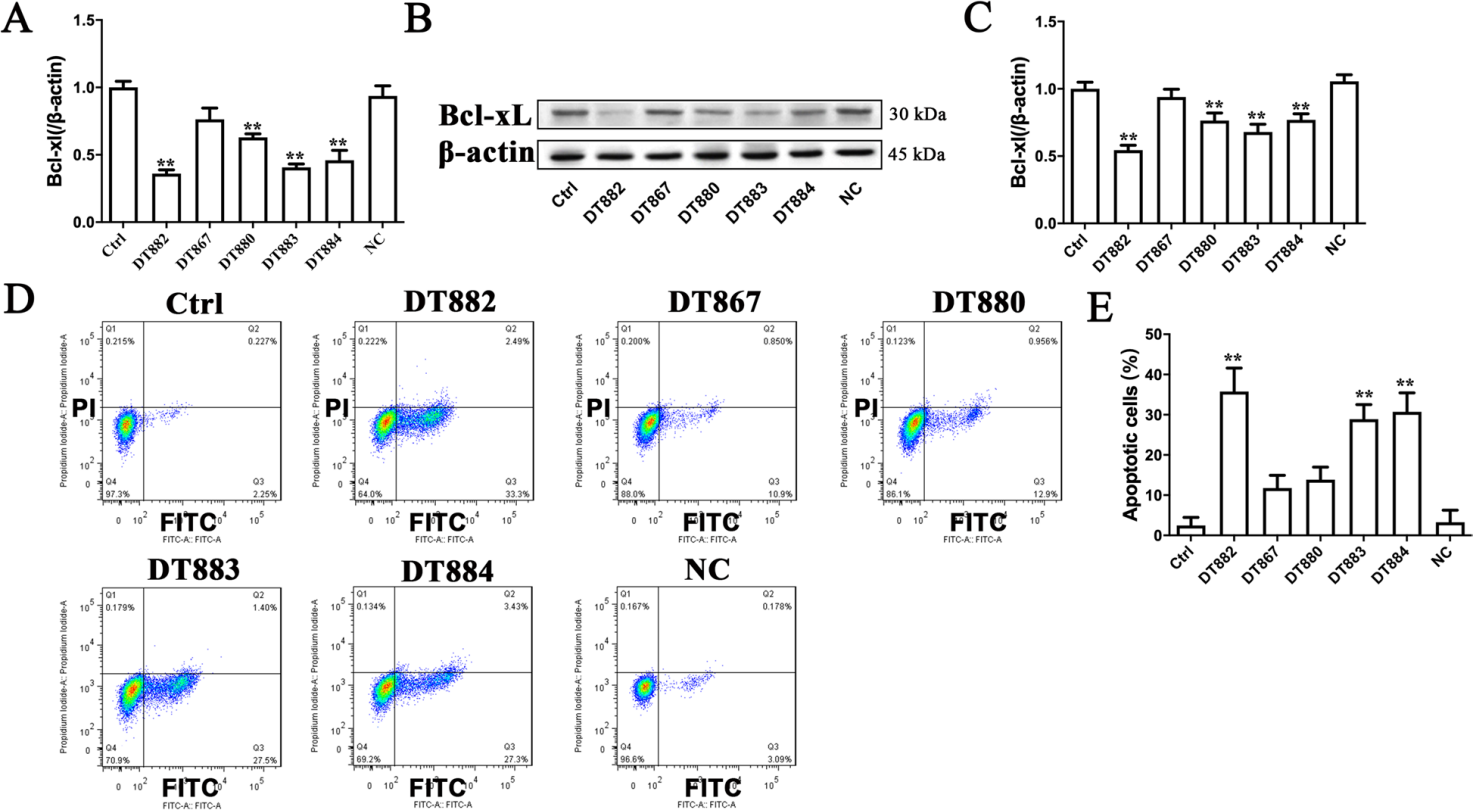
Bcl‑xL DNAzymes inhibited Bcl‑xL expression and caused apoptosis in SW837 cells. After SW837 cells were treated with 4 µM Bcl‑xL DNAzymes for 48 h, Bcl‑xL expression was examined at mRNA (**A**) and protein (**B**-**C**) levels respectively. Apoptosis was detected by Annexin V-FITC/PI assay (**D**), and was expressed as percentages of apoptotic cells (**E**). n = 3. Data were presented as the mean ± SD. *P<0.05, **P<0.01 vs. Ctrl. The cells transfected with a scrambled DNA sequence were negative group (NC). The control cells (Ctrl) did not receive any treatment. Ctrl, control; NC, negative control

DNAzymes are active DNA molecules characterized by binding to and cleaving specific mRNA molecules. In order to determine whether the cytotoxic effects of Bcl‑xL DNAzymes were due to the downregulation of Bcl‑xL, Bcl‑xL expression at both mRNA and protein levels was examined. For SW480 cells, DT882, DT883 and DT884, but not DT867 and DT880, decreased Bcl-xL expression at both mRNA and protein levels (Fig. 3 A-C). DT867, DT880 and the scrambled DNA sequence (negative control) could not reduce the Bcl-xL expression. For SW837 cells, DT880, DT882, DT883 and DT884 decreased Bcl-xL expression at both mRNA and protein levels (Fig. 4 A-C). As an anti-apoptotic factor, Bcl-xL overexpression helps various cancer cells escape from apoptosis. Since DNAzymes could efficiently downregulate Bcl-xL expression, we next investigated whether these DNAzymes caused apoptosis in CRC cells or not. Consistent with the inhibitory effects on Bcl-xL expression, DT882, DT883, and DT884 significantly caused apoptosis in both SW480 and SW837 cells (Figs. 3D-E and 4D-E). Taken together, three DNAzymes (DT882, DT883, and DT884) were identified to have a strong inhibitory effect on Bcl-xL expression. DT882, DT883 and DT884 not only inhibited Bcl‑xL expression, but also caused apoptosis in both SW480 and SW837 cells.

### Either 5-FU or radiotherapy inhibited cell viability, Bcl-xL expression, and apoptosis in SW480 cells

**Fig. 5:**
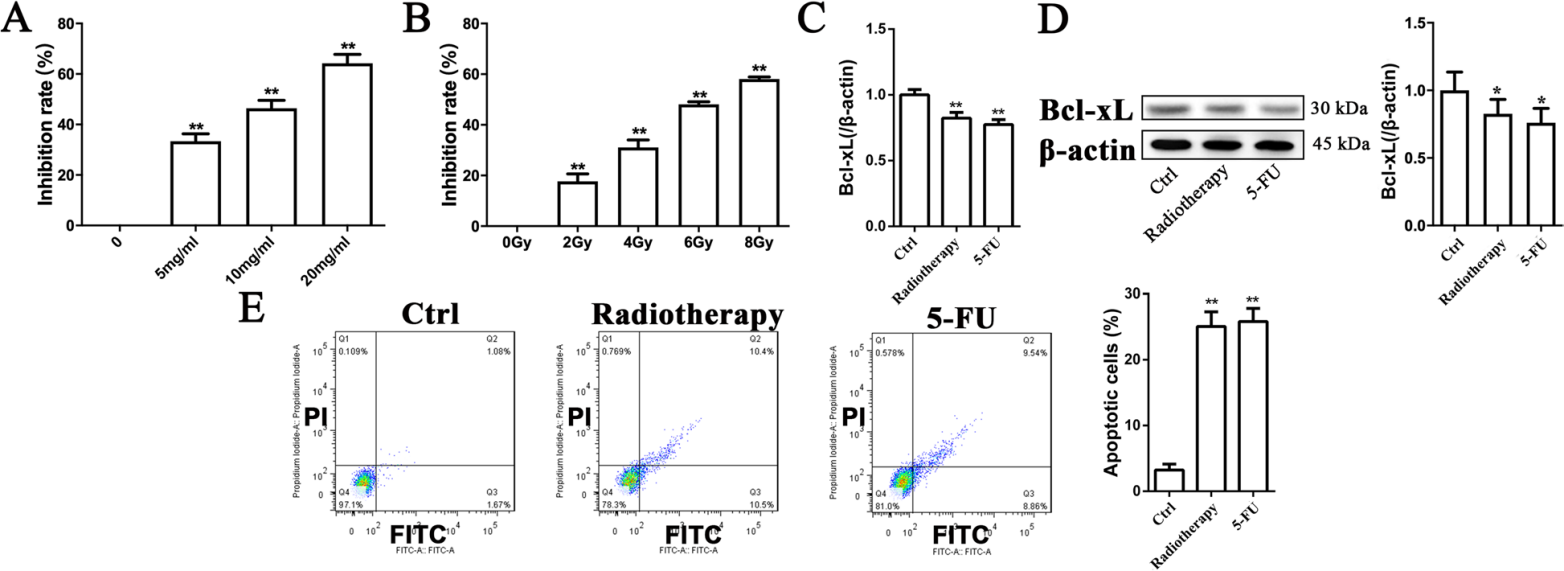
Effects of 5-FU and radiotherapy on cell viability, Bcl-xL expression and apoptosis in SW480 cells. **A**-**B** SW480 cells were treated with 5-FU at 5, 10, 20 mg/mL for 2 days. For radiotherapy, the cells were irradiated with 2, 4, 6, and 8 Gy respectively. Two days after irradiation or incubation with 5-FU, cell viability was detected by MTT method. **C**-**E** 20 mg/ml 5-FU and 8 Gy radiotherapy, which had the highest inhibition rate of cell viability, were used to detect Bcl-xL expression and cell apoptosis. Expression of Bcl-xL mRNA was detected by qPCR assay (**C**). Expression of Bcl-xL protein was detected by Western Blot method (**D**). Apoptosis was detected by Annexin V-FITC/PI assay (**E**). n = 3. Data were presented as the mean ± SD. *P<0.05, **P<0.01 vs. Ctrl. Ctrl, control

Bcl-xL belongs to Bcl-2 family, and has anti-apoptotic effects. Bcl-xL downregulation is closely associated with the cytotoxic effects. Previous studies have suggested that both 5-FU and radiotherapy could lead to apoptosis in CRC cells. In order to investigate whether the downregulation of Bcl-xL was associated with the apoptosis induced by 5-FU or radiotherapy, we measured the cell viability, Bcl-xL expression and apoptosis in CRC cells. As illustrated in Fig. 5 A and B, both 5-FU and radiotherapy significantly inhibited cell viability of SW480 cells in a dose-dependent manner. Based on the above results, 20 mg/ml 5-FU and 8 Gy radiotherapy, which had the highest inhibition rate of cell viability, were chosen for further studies. Both 5-FU and radiotherapy decreased Bcl-xL expression at mRNA and protein levels (Fig. 5 C-D), and caused apoptosis (Fig. 5E). These results suggest that the induction of apoptosis in CRC cells by 5-FU or radiotherapy is associated the suppression of Bcl-xL.

### Radiotherapy or 5-FU combined with Bcl‑xL DNAzyme DT882 enhanced the radiosensitivity and chemosensitivity of SW480 cells *in vitro*

**Fig. 6:**
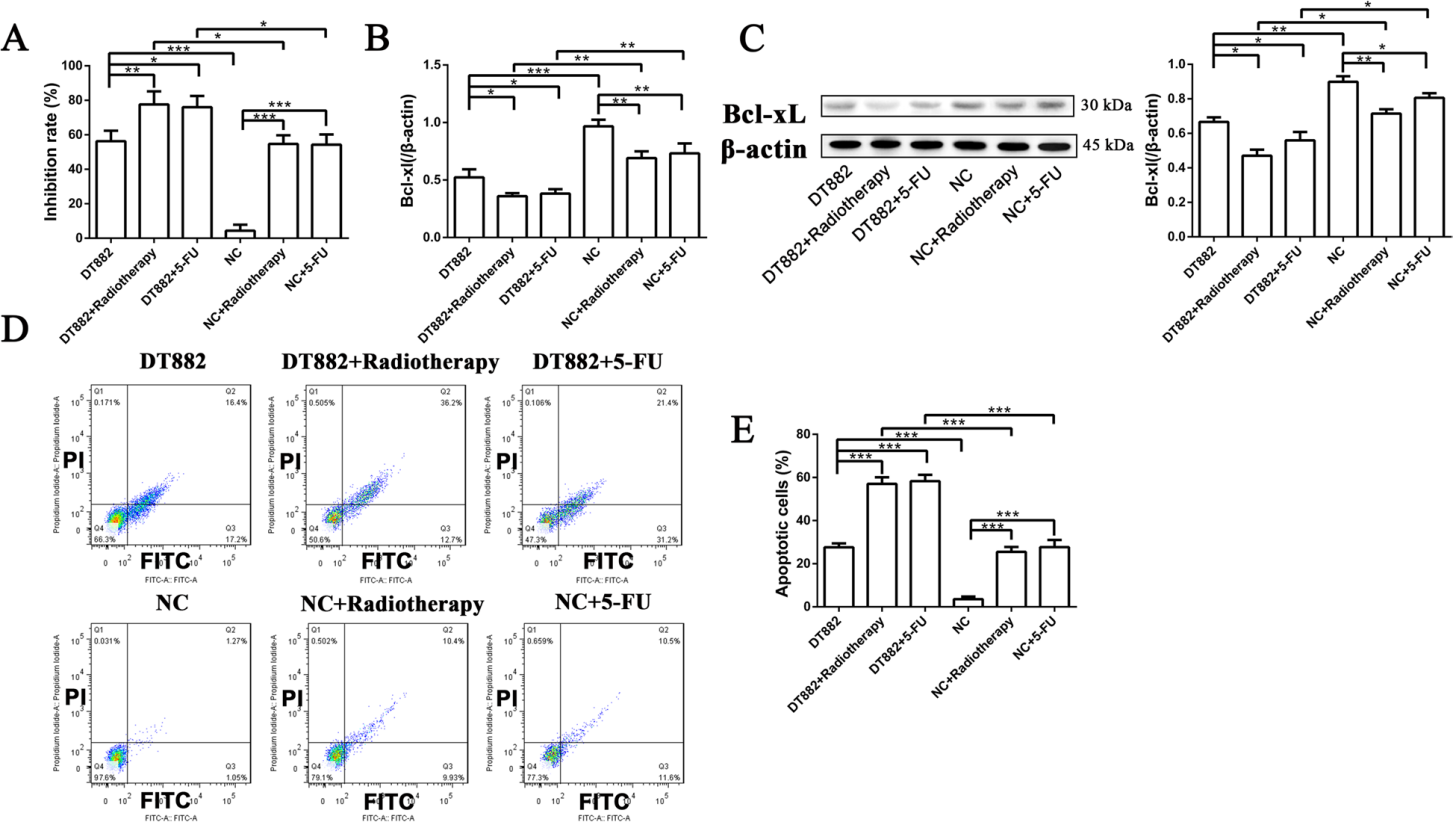
Effects of radiotherapy or 5-FU combined with Bcl‑xL DNAzyme DT882 on cell viability, Bcl-xL expression and apoptosis of SW480 cells in vitro. SW480 cells were first transfected with 4 µM DT882 for 48 h, and then were treated with radiotherapy (8 Gy) or 5-FU (20 mg/mL). After 48 h, the cells were collected. **A** Cell viability was detected by MTT method. **B** Expression of Bcl-xL mRNA was detected by qPCR assay. **C** Expression of Bcl-xL protein was detected by Western Blot method. **D**-**E** Apoptosis was detected by Annexin V-FITC/PI assay. n = 3. Data were presented as the mean ± SD. *P<0.05, **P<0.01, ***P<0.001. Ctrl, control; NC, negative control

Resistance to apoptosis, due to highly expressed Bcl-xL, is considered to play a critical role in chemo- and radiation resistance in CRC cells. Our results showed that both radiotherapy and 5-FU caused apoptosis in CRC cells, accompanied by down-regulation of Bcl‑xL. Targeting Bcl-xL DNAzymes might improve the radiosensitivity and chemosensitivity of CRC cells. Our results demonstrated that radiotherapy or 5-FU in combination with Bcl‑xL DNAzyme DT882 additively increased the inhibition rate of cell viability in SW480 cells (Fig. 6 A; DT882+radiotherapy vs. NC+radiotherapy, DT882+5-FU vs. NC+5-FU). In addition, DT882 additively decreased Bcl-xL expression in either radiotherapy or 5-FU treated cells (Fig. 6B-C). In consistency with the results of cell viability, a significant decrease in apoptosis was observed in the combined treatment group (Fig. 6D-E; DT882+radiotherapy vs. NC+radiotherapy, DT882+5-FU vs. NC+5-FU). These results suggest that combined treatments with radiotherapy (or 5-FU) and Bcl‑xL DNAzyme greatly cause apoptosis. The addictive effects might be associated with the exacerbated down-regulation of Bcl‑xL.

### Radiotherapy or 5-FU combined with Bcl‑xL DNAzyme DT882 reduced the growth of SW480 xenografts *in vivo*

**Fig. 7:**
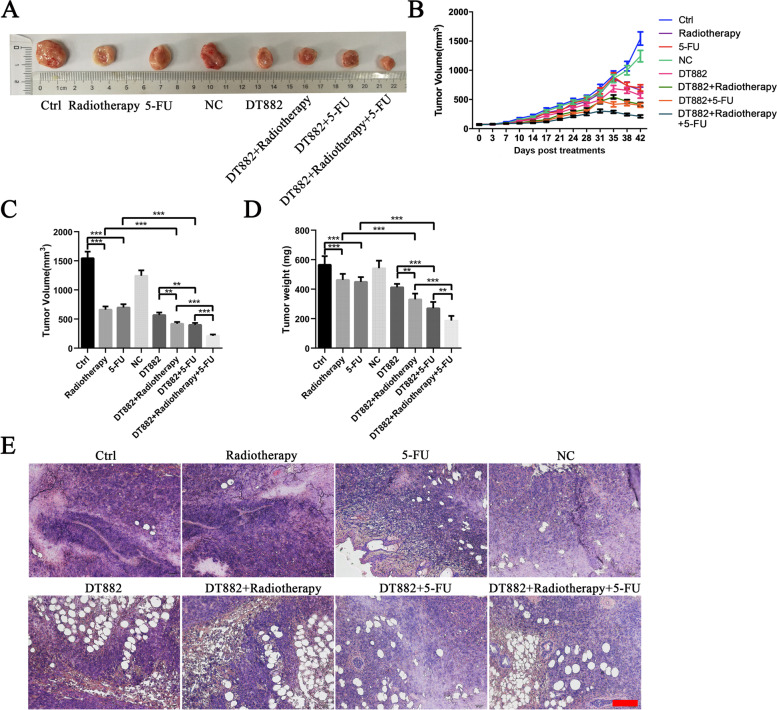
Effects of radiotherapy or 5-FU combined with Bcl‑xL DNAzyme DT882 on the growth of SW480 xenografts *in vivo*. The SW480 xenografts were treated with DNAzymes, 5-FU, radiotherapy or their combinations for 6 weeks. **A** Representative images of excised SW480 xenografts after 6 weeks of treatments. **B** The growth kinetics of tumors among groups. Both the width and length of tumors were measured 2 times per week with a caliper, and the volume was calculated with the formula: volume = length × width × width ×0.5. **C** The tumor volume on day 42. **D** The tumor weight on day 42. **E** Representative images of H&E staining of the tumors. Scale bar: 50 μm. n=6 mice per group. Data were presented as the mean ± SD. **P<0.01, ***P<0.001. Ctrl, control; NC, negative control

We next set out to determine whether radiotherapy (or 5-FU) and Bcl‑xL DNAzyme additively inhibited the growth of CRC *in vivo*. SW480 cells were injected subcutaneously into nude mice to create a mice model with xenograft tumors. The xenografts were then treated with DNAzyme DT882, 5-FU, radiotherapy or their combinations for 6 weeks. After the indicated long term treatments, the tumor volumes in DT882+X-rays group were smaller than the volumes in radiotherapy or DT882 group. Similarly, the tumor volumes in DT882+5-FU group were smaller than the ones in 5-FU or DT882 group. The DT882+radiotherapy+5-FU group was found to have the smallest volume (Fig. 7 A). The growth curves of SW480 xenografts also exhibited the same tendency. The combined treatment with DT882 and 5-FU (or radiotherapy) significantly slowed down the growth rate of SW480 xenografts compared with DT882, 5-FU or radiotherapy alone. The DT882+radiotherapy+5-FU group had the lowest growth rate among the groups (Fig. 7B-D). H&E results showed that DT882, 5-FU or radiotherapy alone slightly caused cell loss in SW480 tumor xenografts, while their combined treatments led to larger areas of cell loss (Fig. 7E). These results suggest that Bcl-xL DNAzymes exacerbate the retarded growth of tumor xenografts treated by DT882, 5-FU or radiotherapy.

## Discussion

In this study, we evaluated whether DNAzymes targeting Bcl-xL could enhance the radiosensitivity and chemosensitivity in CRC cells. DNAzymes targeting Bcl-xL alone caused cell apoptosis in SW837 and SW480 cells via down-regulating Bcl-xL expression. Furthermore, DNAzymes DT882 aggravated the anti-cancer effects of radiotherapy and 5-FU with markedly increased cell apoptosis and decreased Bcl-xL expression. These results suggest that DNAzymes targeting Bcl-xL enhance radiosensitivity and chemosensitivity in CRC cells by causing apoptosis.

Bcl-2 over-expression has been proposed as one of the features of cancers. Therefore, Bcl-2 inhibition is a promising strategy for cancer treatment [23]. Bcl-2 family is well-known for its essential role in the intrinsic apoptotic signalling pathway. Anti-apoptotic Bcl-2 proteins can prevent apoptosis by binding and sequestering the Bcl-2-homology (BH) domains 3-only proteins, and thereby inhibit their interactions with the executioner proteins Bax and Bak [24]. The anti-apoptotic Bcl-2 proteins contains Bcl-2, Bcl-w, Mcl-1, Bfl-1 and Bcl-xL [25]. Compared with other Bcl-2 proteins (Bcl-2 and Mcl-1), Bcl-xL is solely and strongly upregulated in human CRC specimens [11]. If Bcl-xL is specifically knocked-out in intestinal epithelial cells, there is a significantly reduced tumor burden in the inflammation-driven tumor model [11]. When human CRC tissues are treated with ABT-737, an inhibitor for Bcl-xL, an increased number of apoptotic tumor cells is induced [11]. These results suggest that high expression of Bcl-xL plays an important role in CRC occurrence and progression. Apoptosis evasion via Bcl-xL is a promising target for CRC treatment.

DNAzymes are specific sequences of DNA with catalytic activity and can be used as an RNA-cleavage catalyst [12]. For cancer treatment, DNAzymes are usually designed based on cancer markers. Therefore, Bcl-xL mRNA can be used as the target for the design of DNAzymes. Compared with the other strategies reported to inhibit Bcl-xL, such as antisense oligonucleotides, antibodies, peptides or small molecule inhibitors, DNAzymes-based therapy has got more attention, for their catalytic activity is not protein-dependent, and is more selective than siRNA and antisense oligonucleotides [12, 17, 26]. Dozens of DNAzymes have been designed and evaluated in a diverse range of cancers, such as basal cell carcinoma, squamous cell carcinoma, melanoma, breast cancer, prostate cancer, osteosarcoma, liposarcoma, nasopharyngeal carcinoma and colon adenocarcinoma [17]. Among these DNAzymes, Dz13, DZ1, SB010 (hgd40), SB011 (hgd40) and SB012 (hgd40) have been tested in clinical trials [12, 17]. Our previous studies have designed some DNAzymes targeting Bcl-xL, which induce apoptosis in prostate cancer cells PC3 [15]. DT882, DT883 and DT884 could effectively reduce Bcl-xL expression in PC3 cells. DT882 enhances the chemosensitization of multiple cancer cells to Taxol and reverses the resistance of Taxol‑resistance cells CNE2R (nasopharyngeal carcinoma) [19]. In this study, we first examined the effects of these Bcl-xL DNAzymes on cell viability, Bcl-xL expression and apoptosis in CRC cells, and found that DT882, DT883 and DT884 had anti-CRC effects.

At present, 5-FU based chemotherapy and X-rays based radiotherapy are widely used in the clinical treatment of CRC. 5-FU and radiotherapy induced apoptosis in cancer cells is predominantly through the mitochondrial apoptotic pathway [27]. Aberrantly up-regulated expression of anti-apoptotic proteins, such as Bcl-xL, has been found in a wide range of cancers, which confers the resistance to chemotherapy and radiotherapy [25]. Bcl-xL has been clinically demonstrated to be a potential prognostic factor in multiple cancers, including CRC, prostate cancer, non-small cell lung cancer, hepatocellular carcinoma, pancreatic cancer, oropharyngeal cancer, chondrosarcoma and ovarian carcinoma [10, 28–34]. Bcl-xL overexpression is usually associated with poorer prognosis and advanced disease. Bcl-xL is highly expressed in CRC, but not in their adjacent normal mucosa [35]. It has been revealed that high expression of endogenous Bcl-xL is involved in 5-FU resistance. Up-regulation of Bcl-xL inhibits 5-FU-induced apoptosis in CRC cells [36]. Strategies targeting Bcl-xL can effectively enhance the effectiveness of chemotherapy. Antisense oligodeoxynucleotides targeting Bcl-xL have been proved to be effective in leading to apoptosis in CRC cells through down-regulating Bcl-xL expression [35]. miR-122 increases the sensitivity of drug-resistant 5-FU cells via down-regulation of bcl-2 and Bcl-xL [37]. PCDH17 increases the sensitivity of CRC to 5-FU treatment by inducing apoptosis [38]. In this study, we found that Bcl-xL DNAzymes could addictively induce cell apoptosis and reduce tumor growth via down-regulating Bcl-xL. Our results suggest that DNAzymes targeting Bcl-xL are useful for decreasing Bcl-xL expression and inhibiting 5-FU resistance in CRC cells. Further studies are still needed for clinical evaluation.

Like 5-FU resistance in cancer cells, reduced radiosensitivity is closely associated with highly expressed Bcl-xL. Therefore, strategies targeting Bcl-xL are able to enhance the radiosensitivity of cancer cells. siRNA targeting Bcl-xL not only significantly inhibits CRC cell proliferation, migration, and invasion, but also enhances their radiosensitivity by increasing apoptosis [39]. ABT-737, an inhibitor of Bcl-xL, can reverse the acquired radioresistance of breast cancer cells MDA-MB-231 [40]. Radiotherapy combined with ABT-737 dramatically inhibited the tumor growth compared with radiotherapy treatment alone in non-small cell lung tumor xenografts [41]. Combined treatment with ABT-737 and radiotherapy also efficiently increases the radiosensitivity of Hela cells [42]. Bcl-xL down-regulation could significantly enhance the radiosensitivity of osteosarcoma cells and prostate cancer cells [43, 44]. In addition to Bcl-xL inhibitors, Bcl-xL DNAzymes can also achieve the same therapeutic effects as ABT-737. Our study demonstrated that irradiation by X-rays decreased Bcl-xL expression in CRC cells, suggesting that Bcl-xL down-regulation might participate in irradiation induced apoptosis. When radiotherapy was combined with Bcl-xL DNAzymes, apoptosis was significantly induced. Our results suggest that DNAzymes treatment combined with conventional radiotherapy may be an effective therapeutic strategy for future treatment of CRC.

It should be noted that the underlying mechanisms of Bcl-xL DNAzymes are different from Bcl-xL inhibitors. The extensively studied ABT-737 is a BH3 mimetic drug. ABT-737 can bind to Bcl-2 proteins with high affinity as an antagonist, and then disrupts the bindings of Bcl-2 proteins with Bax and Bak, thereby inducing cell apoptosis [45]. In contrast, the catalytic activity of DNAzymes is not dependent on proteins. DNAzymes have a high selectivity in the recognition of targeted sequences. DNAzymes alone can selectively bind to the mRNA of the targeted proteins, and inhibit their translation directly [12, 15]. Furthermore, DNA is much cheaper and more stable than other types of drugs. These advantages make DNAzymes a promising strategy for molecular targeted therapy. Bcl-xL DNAzymes DT882, DT883, and DT884, identified in this study may have prospects of clinical application.

## Conclusions

Therapeutic agents targeting Bcl-xL by DNAzymes could enhance the pro-apoptotic potential of chemotherapy or radiotherapy. Here, we identified three DNAzymes targeting Bcl-xL, DT882, DT883 and DT884. These Bcl-xL DNAzymes effectively reduced Bcl-xL expression and caused cell apoptosis in SW480 and SW837 cells. Either 5-FU or radiotherapy induced cell apoptosis and decreased Bcl-xL expression. When combined with 5-FU or radiotherapy, Bcl-xL DNAzymes addictively promoted apoptosis by down-regulating Bcl-xL *in vitro*, and significantly inhibited the growth of SW480 xenograft. Overall, our studies suggest that DNAzymes downregulate Bcl-xL expression and sensitize CRC cells to 5-FU and radiotherapy. Our study provides an alternative therapeutic strategy for the treatment of CRC.

## Supplementary Information

**Additional file 1.**

## Data Availability

The datasets used and/or analyzed during the current study are available from the corresponding author on reasonable request.

## Abbreviations

5-FU: 5-fluorouracil
Bcl-xL: B-cell lymphoma-extra large
CRC: Colorectal cancer.
DNAzymes: Deoxyribozymes
