# Supplementary material for: Bcl-xL DNAzymes promote radiosensitivity and chemosensitivity in colorectal cancer cells via enhancing apoptosis
Source: BMC Pharmacol Toxicol. 2022 Feb 5;23:13. doi: 10.1186/s40360-022-00553-x (PMC8817578; doi:10.1186/s40360-022-00553-x)
Supplement: Supplementary file 1 — Additional file 1. [file 40360_2022_553_MOESM1_ESM.docx]

The original and different exposure images of WB results.

**Figure 3B:** Effects of Bcl‑xL DNAzymes on protein levels of Bcl‑xL in SW480 cells.

Gel of Bcl-xL:


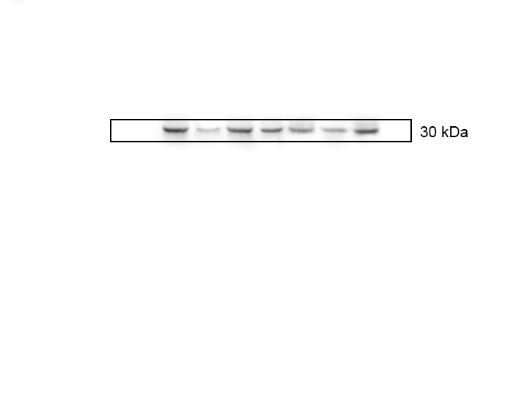

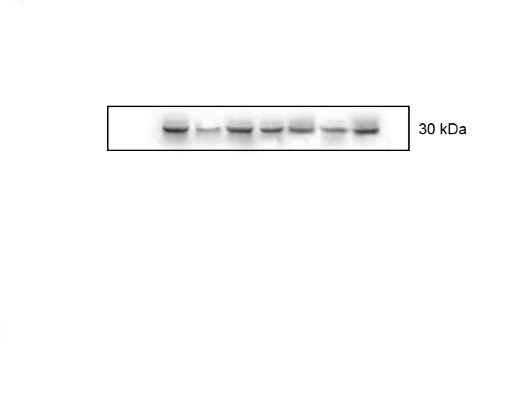

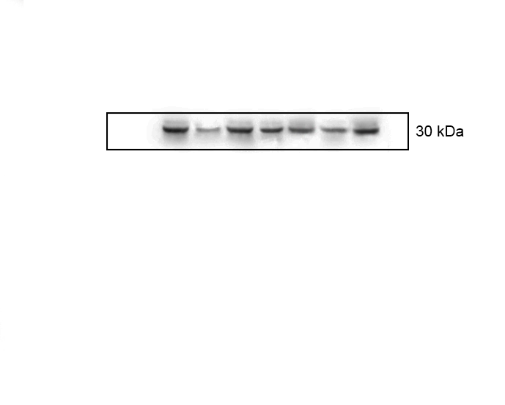


Gel of β-actin:


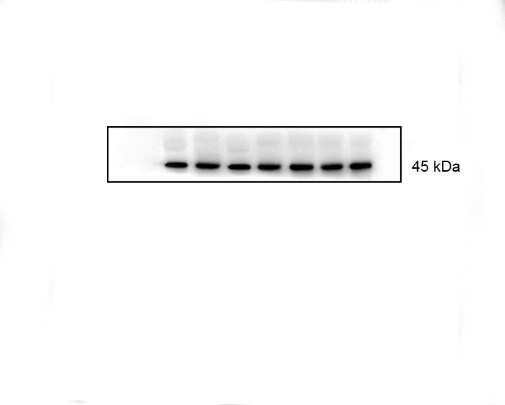

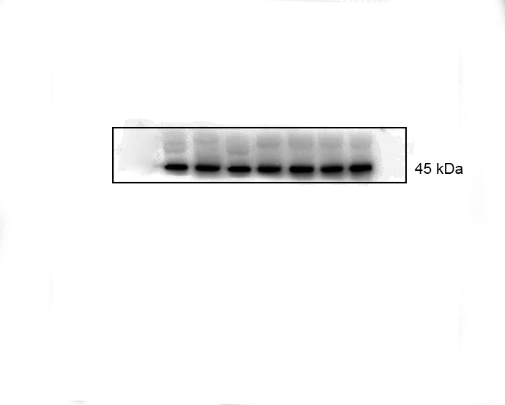

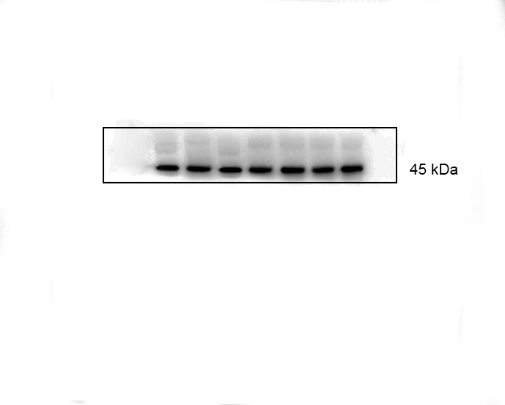


These lanes from left to right are Ctrl, DT882, DT867, DT880, DT883, DT884 and NC.

**Figure 4B:** Effects of Bcl‑xL DNAzymes on protein levels of Bcl‑xL in SW837 cells.

Gel of Bcl-xL:


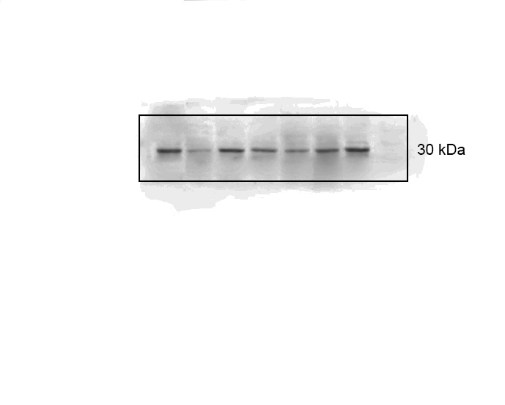

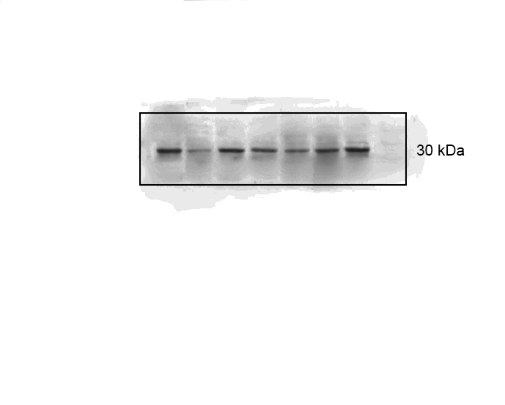

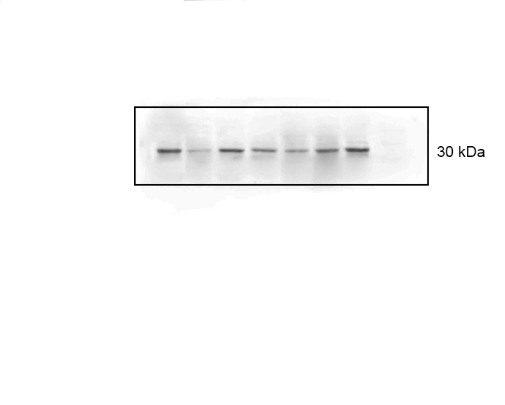


Gel of β-actin:


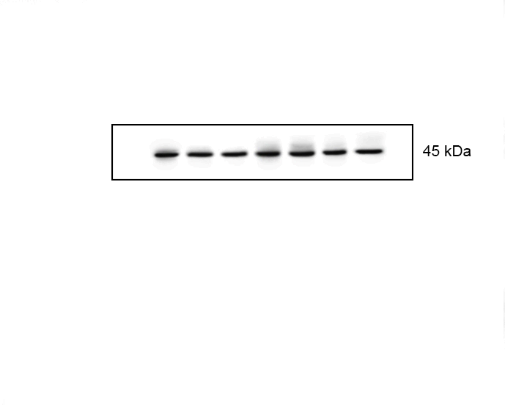

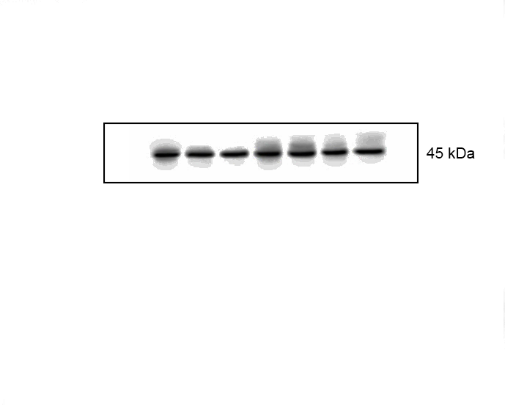

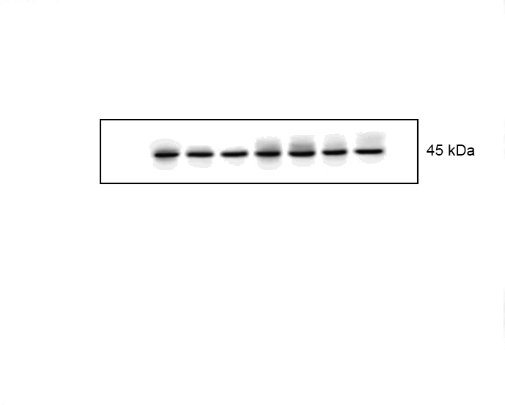


These lanes from left to right are Ctrl, DT882, DT867, DT880, DT883, DT884 and NC.

**Figure 5D:** Effects of 5-FU and radiotherapy on Bcl-xL protein levels in SW480 cells.

Gel of Bcl-xL:


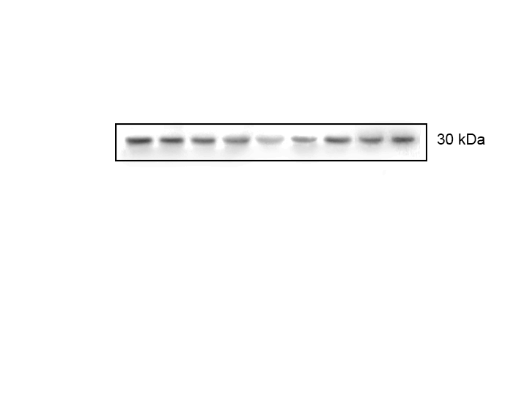

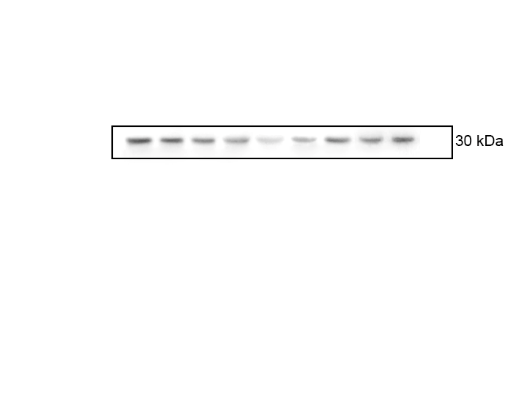

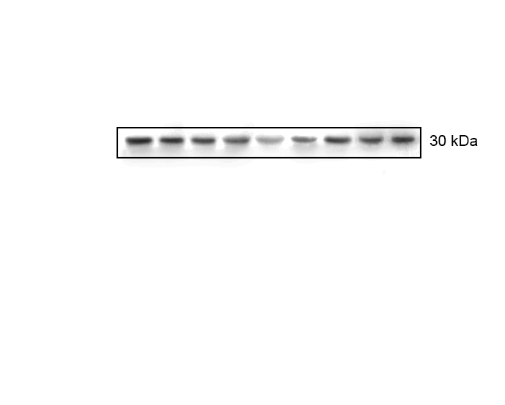


Gel of β-actin:


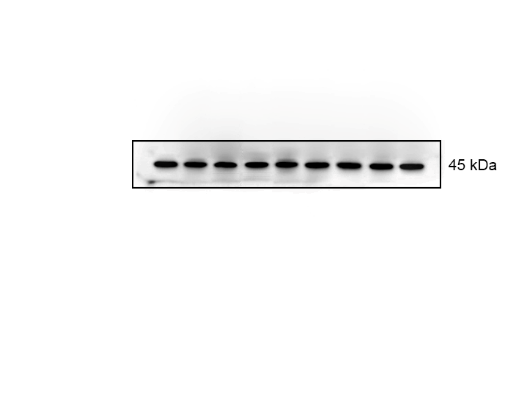



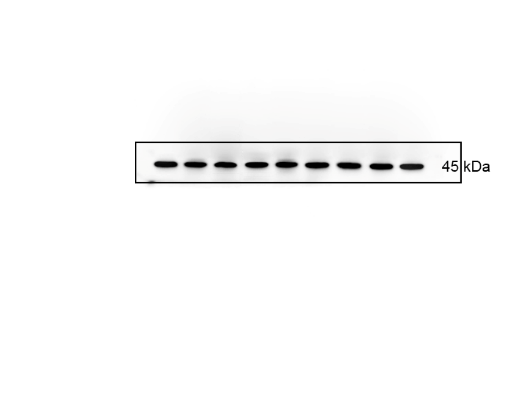


The first three lanes were used in Figure 5D. These lanes from left to right are Ctrl, Radiotherapy and 5-FU.

**Figure 6C:** Effects of radiotherapy or 5-FU combined with Bcl‑xL DNAzyme DT882 on Bcl-xL protein levels in SW480 cells.

Gel of Bcl-xL:


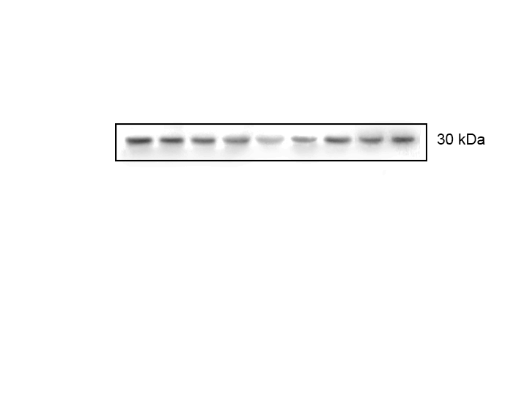

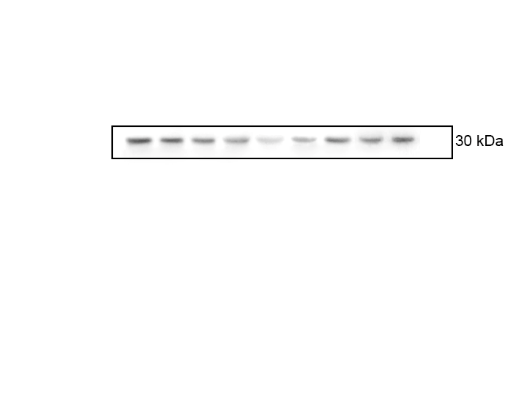

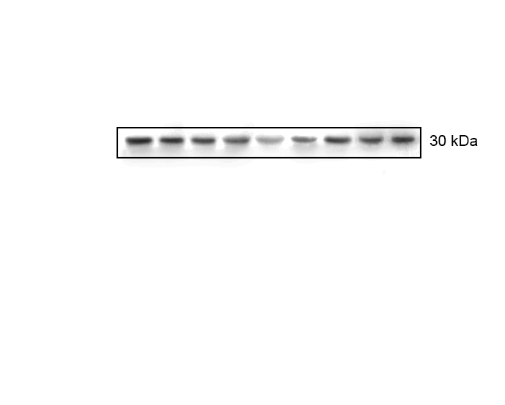


Gel of β-actin:


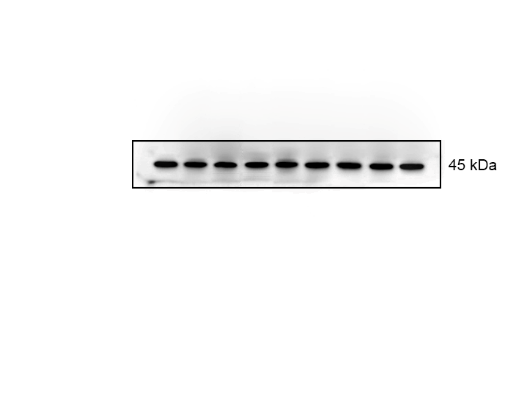



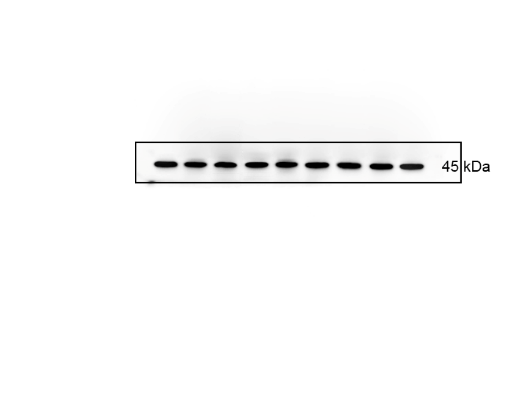


The lanes 4 to 9 from left to right were used in Figure 6C. These lanes from left to right are DT882, DT882+Radiotherapy, DT882+5-FU, NC, NC+Radiotherapy, and NC+5-FU.
